# Naples Prognostic Score and Clinical Outcomes After PCI for Acute Coronary Syndrome: A Systematic Review and Meta‐Analysis

**DOI:** 10.1002/clc.70247

**Published:** 2025-12-29

**Authors:** Yasin Özen, Mustafa Bilal Ozbay, Zahin Shahriar, Hüseyin Tezcan, Tugay Dedebali, Abdullah Tunçez, Muhammed Ulvi Yalçin, Kadri Murat Gürses, Bülent Özbay

**Affiliations:** ^1^ Department of Cardiology Konya Selcuk University Medical Faculty Hospital Konya Turkey; ^2^ Department of Medicine Penn Medicine Princeton Medical Center Plainsboro New Jersey USA; ^3^ Department of Cardiology Dhaka Medical College Hospital Dhaka Bangladesh; ^4^ Department of Pulmonary Medicine, Faculty of Medicine Muğla Sıtkı Kocman University Muğla Turkey

## Abstract

**Background:**

Naples Prognostic Score (NPS), a composite index incorporating inflammatory and nutritional markers, has emerged as a potential prognostic tool in various cardiovascular conditions; however, no meta‐analysis has yet pooled the available evidence to comprehensively assess its prognostic utility.

**Objectives:**

To evaluate the association of NPS with clinical outcomes, including all‐cause mortality, in‐hospital mortality, no‐reflow (NR) phenomenon, and left ventricular ejection fraction (LVEF), in acute coronary syndrome (ACS) patients undergoing percutaneous coronary intervention (PCI).

**Methods:**

MEDLINE, Cochrane, and EMBASE databases were searched for studies comparing high and low NPS groups in ACS patients undergoing PCI. Random‐effects models were used to pool risk ratios (RR) for binary outcomes and mean differences (MD) for continuous outcomes. Heterogeneity was assessed with I² statistics. Statistical analyses were performed using Review Manager 5.4, and R, version 4.2.2.

**Results:**

We included seven studies comprising 13 268 patients, with 5628 (42.4%) patients in the low NPS group. Low NPS was significantly associated with decreased all‐cause mortality (RR: 0.42; 95% CI: 0.32–0.55; I² = 48%) and decreased incidence of NR (RR: 0.60; 95% CI: 0.40–0.88; I² = 83%). Patients with low NPS also had higher LVEF (MD: −2.69%; 95% CI: −3.41 to −1.97; I² = 99%). No significant difference was observed in in‐hospital mortality (RR: 0.54; 95% CI: 0.28–1.05; I² = 94%).

**Conclusion:**

In ACS patients undergoing PCI, elevated NPS was associated with worse clinical outcomes. These findings support the use of NPS as a practical, biomarker‐based tool for risk stratification in this population.

## Graphical abstract

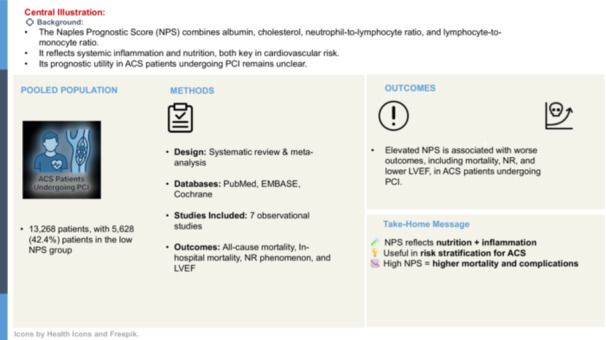

## 1 Introduction

Acute coronary syndrome (ACS) remains a leading cause of morbidity and mortality worldwide despite advancements in percutaneous coronary intervention (PCI) techniques and adjunctive pharmacologic therapies [1, 2]. Timely risk stratification plays a major role in guiding therapeutic decisions and optimizing clinical outcomes in this high‐risk population [3, 4]. Although traditional risk assessment models primarily focus on clinical and angiographic variables, there is increasing interest in integrating inflammatory and nutritional biomarkers to enhance prognostic accuracy [5, 6].

The Naples Prognostic Score (NPS) is a novel biomarker‐based scoring system that incorporates serum albumin, total cholesterol, neutrophil‐to‐lymphocyte ratio (NLR), and lymphocyte‐to‐monocyte ratio (LMR). Originally developed to predict outcomes in oncology and chronic illnesses, recent studies have explored its prognostic value in cardiovascular diseases, including ACS [7, 8]. Given its composite nature, the NPS may reflect both systemic inflammation and nutritional status, two factors that are closely linked to adverse cardiovascular events [5, 9].

Several observational studies have assessed the prognostic value of the NPS in patients undergoing PCI for ACS. However, these studies have yielded heterogeneous findings, and most are limited by relatively small sample sizes [7, 10, 11, 12, 13, 14, 15]. To address these limitations and provide a more precise estimation of the prognostic relevance of NPS, we conducted a systematic review and meta‐analysis of the available evidence. This study aimed to compare clinical outcomes, including all‐cause mortality, in‐hospital mortality, NR, and LVEF, between patients with high and low NPS undergoing PCI for ACS.

## 2 Methods

This study was designed in accordance with the Preferred Reporting Items for Systematic Reviews and Meta‐Analysis protocol by the Meta‐analysis of Observational Studies in Epidemiology (MOOSE) statement and Preferred Reporting Items for Systematic review and Meta‐analysis (PRISMA) 2020 checklist [16, 17]. The protocol was prospectively registered in the International Prospective Register of Systematic Reviews (PROSPERO) database with the registration number CRD420251066172.

### 2.1 Search Strategy

MEDLINE, Cochrane and EMBASE databases were systematically searched from inception to June 2025. References for eligible papers and systematic reviews were also searched for additional studies of interest. The complete search strategy for each database can be found in the Supporting Information.

### 2.2 Eligibility Criteria and Data Extraction

Inclusion criteria of this meta‐analysis were [1]: non‐randomized prospective or retrospective design [2]; studies comparing clinical outcomes between patients with low and high NPS undergoing PCI for ACS and [3] reporting at least one of the following clinical endpoints: all‐cause mortality, in‐hospital mortality, NR, or LVEF. We excluded studies with overlapping patient populations, studies published only as conference abstracts, case or case series reports, or studies published in languages other than English.

Three investigators independently conducted the literature search and study selection. Discrepancies were resolved through consensus following a full‐text review and reassessment of the eligibility criteria, in consultation with the senior author. Data extraction was also performed independently by the same three investigators, and all extracted data were verified by the senior author for accuracy and consistency. Variability in reporting across included studies resulted in selective data availability for certain baseline characteristics. When a study did not provide a specific variable for either the low or high NPS subgroup, the value was recorded as “NA” to accurately reflect the absence of reporting rather than missing data within our extraction process.

### 2.3 Endpoints

Included outcomes were: all‐cause mortality, in‐hospital mortality, NR phenomenon, or LVEF. Additionally, all studies were required to stratify patients based on high and low NPS. Patients were stratified into low (NPS 0–2) and high (NPS 3–4) groups based on the NPS, which incorporates serum albumin, total cholesterol, NLR, and LMR values, as described in the Supporting Information S1: Table 1.

### 2.4 Risk of Bias Assessment

The Risk Of Bias in Non‐randomized Studies of Interventions (ROBINS‐I) tool was used to evaluate the non‐RCTs studies, with seven domains: confounding, selection of participants, classification of interventions, deviations from intended interventions, missing data, measurement of outcomes, and reported result [18]. Two independent investigators evaluated each included study, and any discrepancies were resolved in consultation with the senior author. A funnel plot was not generated because the number of included studies was fewer than 10, which limits the reliability of publication bias assessment [19].

### 2.5 Data Analysis

We conducted all data analysis following the recommendations outlined by Cochrane [20]. Binary endpoints were summarized using the Mantel‐Haenszel test with a random effects model risk ratio (RR) and 95% confidence interval (CI). Continuous endpoints were summarized using mean difference (MD) and 95% CI. We assessed the heterogeneity using Cochrane's Q statistic and Higgins and Thompsons’ I^2^ statistic. P values inferior to 0.10 and I^2^ > 50% were considered significant for heterogeneity. We performed sensitivity analyses using the “leave‐one‐out” approach. Statistical analyses were performed using Review Manager 5.4 (The Nordic Cochrane Centre, The Cochrane Collaboration, Denmark) and R, version 4.2.2 (R Foundation for Statistical Computing) using the metafor package.

## 3 Results

### 3.1 Study Selection and Characteristics

**FIGURE 1:**
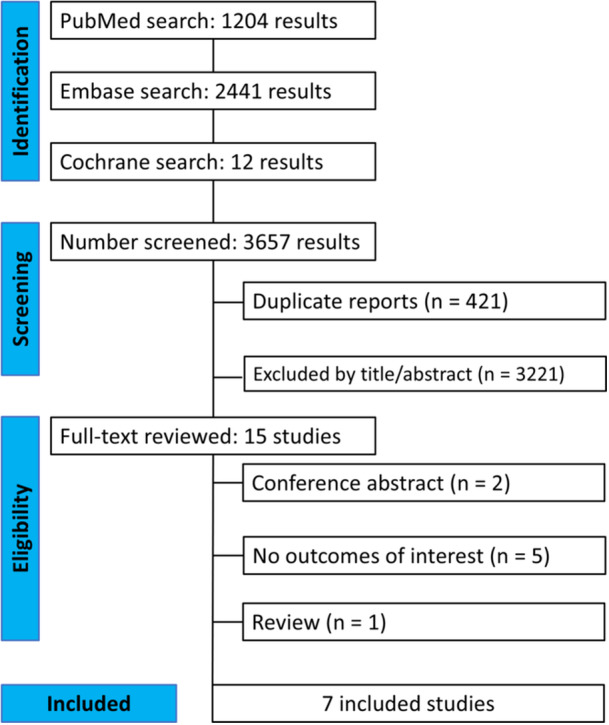
PRISMA flow diagram of study screening and selection.

As illustrated in Figure 1, the initial database search identified 3657 records. Following the removal of duplicates and screening based on titles and/or abstracts, 15 studies were selected for full‐text review. Of these, seven studies met the predefined inclusion criteria [7, 10, 11, 12, 13, 14, 15]. All seven included studies were conducted in Turkey, resulting in a geographically and demographically homogeneous study population. This systematic review and meta‐analysis included a total of 13 268 patients, with 5628 (42.4%) patients classified into the low NPS group and 7640 (57.6%) patients into the high NPS group. The mean age across studies ranged from 55.5 to 66.0 years. The proportion of male participants varied between 52.0% and 87.8%. Follow‐up duration differed among studies, including in‐hospital follow‐up, 1‐year follow‐up, and up to 8 years in one study [10]. Five studies included patients with acute ST‐segment elevation myocardial infarction (STEMI) [7, 10, 11, 12, 15], one study included patients with non‐ST‐segment elevation myocardial infarction (NSTEMI) [14], and one focused on patients undergoing PCI of saphenous vein graft (SVG) for ACS [13]. Additional details about the characteristics of the included studies are provided in Table 1.

**TABLE 1:** Study characteristics.

	Birdal, 2024 (*N* = 2280)		Erdogan, 2023 (*N* = 1887)		Gitmez, 2025 (*N* = 197)		Karakoyun, 2024 (*N* = 2901)		Saygi, 2024 (*N* = 3828)		Saylik, 2023 (*N* = 1889)		Yilmaz, 2024 (*N* = 286)	
Characteristics	Low NPS (*n* = 799)	High NPS (*n* = 1481)	Low NPS (*n* = 739)	High NPS (*n* = 1148)	Low NPS (*n* = 111)	High NPS (*n* = 86)	Low NPS (*n* = 1012)	High NPS (*n* = 1889)	Low NPS (*n* = 2004)	High NPS (*n* = 1824)	Low NPS (*n* = 824)	High NPS (*n* = 1065)	Low NPS (*n* = 139)	High NPS (*n* = 147)
Follow‐up duration	No follow‐up		NA		1 year		In‐hospital		In‐hospital		8 years^b^		In‐hospital	
Population	STEMI		STEMI		NSTEMI		STEMI		STEMI		STEMI		ACS	
Male, %	75.5	77.9	79.2	76.0	64.0	52.0	81.2	76.4	80.0	75.9	69.8	70.8	83.5	87.8
Age, years^a^	58.2	59.2	56.0	62.0	61.3	64.9	55.5	60.0	57.0	59.7	64.0	64.0	66.0	64.0
Diabetes mellitus, %	22.7	24.6	26.5	30.7	35.0	38.0	21.3	23.3	25.0	24.5	21.1	24.2	49.6	44.9
Hypertension, %	43.3	42.9	42.9	49.1	50.0	52.0	41.7	43.0	45.0	45.0	38.0	45.4	81.3	76.0
HLP, %	40.4	38.9	4.6	8.6	16.0	8.0	53.4	31.9	12.0	17.5	32.5	47.0	77.7	74.1
Multivessel disease, %	41.8	42.9	39.4	48.3	NA	NA	NA	NA	38.7	38.5	NA	NA	100.0	100.0
Anterior MI a	47.4	47.9	42.6	41.2	0	0	45.9	49.4	42.0	48.2	38.6	42.3	NA	NA
Previous MI^a^	NA	NA	16.6	15.8	NA	NA	NA	NA	13.0	13.0	NA	NA	NA	NA
Previous CABG^a^	0	0	NA	NA	0	0	NA	NA	4.0	2.7	NA	NA	100.0	100.0
LVEF, %	47.7	45.6	50.0	45.0	NA	NA	47.9	46.0	48.0	46.1	44.7	44.0	65.0	55.0

Abbreviations: ACS, acute coronary syndrome; CABG, coronary artery bypass graft; HLP, hyperlipidemia; LVEF, left ventricular ejection fraction; MI, myocardial infarction; NSTEMI, non‐ST‐elevation myocardial infarction; NA, non‐available; NPS, Naples prognostic score; STEMI, ST‐elevation myocardial infarction.

^a^ Mean or median.

^b^ The maximum follow‐up was 8 years.

### 3.2 Pooled Analysis of Outcomes

**FIGURE 2:**
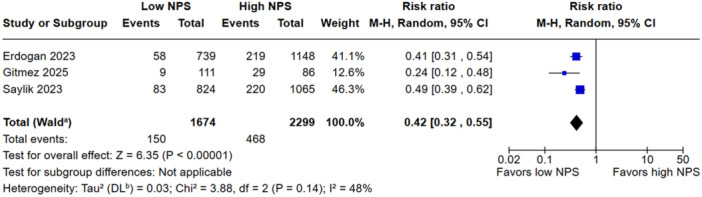
The incidence of all‐cause mortality was significantly reduced in patients with low NPS.

**FIGURE 3:**
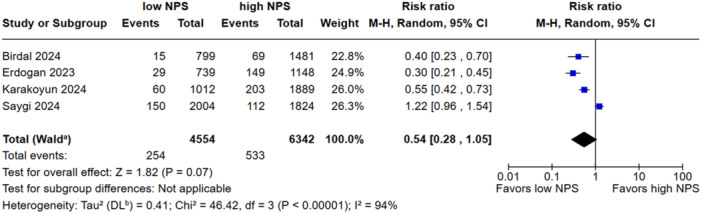
The incidence of in‐hospital mortality did not differ between the low and high NPS groups.

**FIGURE 4:**
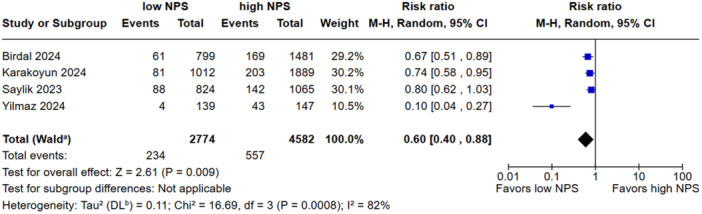
The incidence of no‐reflow was significantly decreased in patients with low NPS.

**FIGURE 5:**
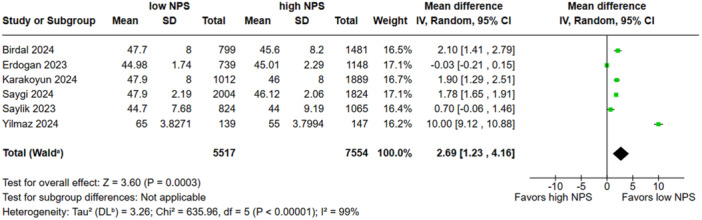
Patients with high NPS had a significantly lower left ventricular ejection fraction (LVEF) by 2.69% compared to those with low NPS.

Low NPS was associated with a reduced all‐cause mortality when compared to the high group (RR, 0.42; 95% CI, 0.32–0.55; *p* < 0.00001; I^2^ = 48%; Figure 2). There was no significant difference in in‐hospital mortality between groups (RR, 0.54; 95% CI, 0.28–1.05; *p* = 0.07 I^2^ = 94%; Figure 3). Low NPS group was associated with a decreased rate of NR when compared to the high group (RR, 0.60; 95% CI, 0.40–0.88; *p* = 0.009 I^2^ = 83%; Figure 4). Patients with a low NPS had a significantly higher LVEF by a mean difference of 2.69% (95% CI: −3.41 to −1.97; I² = 99%; Figure 5). The overall risk of bias in observational studies was rated as moderate (Supporting Information S1: Table 4).

### 3.3 Sensitivity Analysis

In the sensitivity analysis, the overall effect size for all‐cause mortality was robust, with no single study exerting a disproportionate influence; the pooled estimate (RR: 0.42; 95% CI: 0.32–0.55) and heterogeneity (I² = 48.4%) remained stable. In‐hospital mortality showed a nonsignificant overall association (RR: 0.54; 95% CI: 0.28–1.05; I² = 94%), although excluding Saygi et al. 2024 reduced heterogeneity to 68.2% and yielded a significant effect (RR: 0.41; 95% CI: 0.28–0.62). For the NR outcome, the effect size remained stable (RR: 0.60; 95% CI: 0.40–0.88; I² = 82.0%); however, omitting Yilmaz et al. 2024 led to a marked reduction in heterogeneity (I² = 0%) with preserved significance (RR: 0.74; 95% CI: 0.64–0.86). Regarding LVEF, the pooled MD remained consistent (MD: 2.69; 95% CI: 1.23–4.16; I² = 99.2%) across all iterations. Leave‐one‐out analyses are presented in Supporting Information S1: Figures 1–4.

## 4 Discussion

In this systematic review and meta‐analysis comprising seven studies [7, 10, 11, 12, 13, 14, 15] and more than 13 000 patients, we compared patients with low and high NPS undergoing PCI for ACS. The main findings were as follows [1]: low NPS was associated with a decreased risk of all‐cause mortality and NR phenomenon [2]; significantly lower LVEF in patients with high NPS; and [3] no statistically significant difference in in‐hospital mortality between the two groups.

These findings highlight the potential utility of NPS as a non‐invasive, readily available tool for prognostic risk stratification in ACS. Given that NPS integrates nutritional and inflammatory markers, its association with prognostic outcomes is noteworthy [15, 20, 21]. This likely reflects the contribution of systemic inflammation and malnutrition to atherosclerosis progression and myocardial injury burden [22, 23]. Previous studies have established the prognostic significance of each of these individual parameters in cardiovascular disease [24, 25, 26]. To our knowledge, this is the first meta‐analysis to comprehensively evaluate the prognostic utility of the NPS in ACS patients undergoing PCI.

The observed association between high NPS and increased risk of NR aligns with the hypothesis that elevated systemic inflammation may exacerbate microvascular dysfunction and distal embolization during PCI [27, 28]. Our leave‐one‐out sensitivity analysis demonstrated that omitting Yilmaz et al. [13] substantially reduced heterogeneity while maintaining the statistical significance of the association. Notably, this study [13] was the only one to focus on patients undergoing PCI for SVG lesions, a population known to have distinct procedural challenges and higher NR rates [29]. PCI of SVGs is consistently associated with a substantially higher risk of the NR phenomenon compared with native coronary arteries, owing to their diffuse, friable, lipid‐rich plaques, large thrombus burden, and high propensity for distal embolization [29]. This may partly explain the substantial heterogeneity observed in the NR analysis. However, even after omitting Yilmaz et al. [13], low NPS remained associated with a reduced incidence of no‐reflow, indicating the robustness of the pooled estimates.

Similarly, lower LVEF among patients with high NPS may reflect a more advanced inflammatory state and myocardial injury burden, supporting the pathophysiological link between inflammation, malnutrition, and left ventricular (LV) dysfunction [30, 31]. Although the pooled analysis demonstrated a statistically significant reduction in LVEF among patients with high NPS (MD: −2.69%; 95% CI: −3.41 to −1.97), the clinical relevance of this difference warrants careful consideration. In routine cardiology practice, changes in LVEF of ≥ 5% are typically regarded as clinically meaningful, raising the possibility that the observed 2.69% reduction, while statistically significant, may have limited direct clinical impact [32, 33]. Additionally, the substantial heterogeneity observed for this outcome (I² = 99%) suggests variability in measurement methods, patient characteristics, or timing of LVEF assessment across studies. Therefore, while the direction of effect aligns with the hypothesis that elevated inflammation and poor nutritional status are associated with worse ventricular function, the modest absolute difference should be interpreted cautiously.

In contrast to the association between high NPS and all‐cause mortality, our pooled analysis did not demonstrate a statistically significant difference in in‐hospital mortality between patients with high and low NPS. Several factors may explain this finding. First, in‐hospital mortality is influenced by acute events, procedural complications, and immediate post‐intervention care, which may not be fully captured by a prognostic index like the NPS, which reflects chronic inflammation and nutritional status [34, 35]. Therefore, the short time frame may limit the discriminatory power of NPS for in‐hospital outcomes. Additionally, the limited number of studies reporting this endpoint (only four of the seven included studies reported in‐hospital mortality rates), combined with the relatively low event rates, may have diminished the statistical power to detect a significant difference [7, 11, 12, 15].

In addition, in the analysis of in‐hospital mortality, the study by Saygi et al. [15] appeared to contribute disproportionately to the observed heterogeneity. Sensitivity analysis excluding the study by Saygi et al. [15] substantially reduced heterogeneity (I² = 68.2%) and resulted in a statistically significant association (RR: 0.41; 95% CI: 0.28–0.62). This may be explained by several differences compared with the other included studies. Saygi et al. used a different way of grouping patients according to NPS such as three‐tier categorization (NPS 0–2, 3, and 4) and included patients from an earlier study period compared to other studies. In addition, patients with higher NPS in this study had more severe clinical features, such as older age, worse cardiac function, and higher inflammatory markers, all of which are known to increase short‐term mortality risk. In their study, no significant difference in in‐hospital outcomes was observed between the low and high NPS groups. However, when patients were stratified into four categories, those with a NPS of 4 exhibited higher in‐hospital mortality. Furthermore, a Naples score of 4 was independently associated with an increased risk of in‐hospital mortality, as demonstrated by multivariate analysis (adjusted odds ratio: 2.83; 95% CI: 1.76–4.54).

To contextualize the clinical relevance of NPS, its discriminatory performance should be considered alongside established risk scores such as TIMI and GRACE, which remain the most widely validated tools for prognostication in ACS [36]. The TIMI score, introduced by Antman et al. [37], stratifies risk based on simple bedside variables and predicts short‐term mortality and ischemic events. Similarly, the GRACE score incorporates hemodynamics and laboratory parameters to estimate in‐hospital and long‐term mortality [38]. Unlike these scores, which rely primarily on clinical and hemodynamic predictors, the NPS captures nutritional and inflammatory status, offering complementary prognostic information that may refine risk stratification, particularly for identifying patients vulnerable to no‐reflow or adverse remodeling. None of the included studies systematically compares NPS directly with GRACE or TIMI; therefore, our meta‐analysis cannot quantify the incremental predictive value of NPS over guideline‐recommended scores. Future studies directly comparing NPS with TIMI and GRACE in ACS populations are needed to determine whether it meaningfully improves risk prediction beyond these validated tools.

The results of this meta‐analysis suggest that the NPS can be feasibly incorporated into routine ACS workflows because it relies solely on laboratory parameters already obtained at presentation [5, 9]. As a rapid, cost‐neutral tool, the NPS may complement existing risk scores by identifying patients with increased inflammatory and nutritional vulnerability who are at increased risk for no‐reflow, lower LVEF, and long‐term mortality. Clinically, patients with high NPS may warrant closer monitoring during PCI, more intensive secondary prevention strategies, and targeted nutritional or inflammatory optimization after discharge.

Because the NPS is composed entirely of laboratory markers routinely obtained at admission, it should be calculated at the time of initial ACS presentation, before PCI, to support early risk stratification and procedural planning. Patients with elevated NPS may benefit from enhanced periprocedural monitoring, anticipatory measures for no‐reflow risk, and intensified secondary prevention strategies. In select patients with ongoing inflammatory activity, hemodynamic instability, or nutritional decline during hospitalization, repeating NPS may offer additional prognostic value. Until validated across diverse international cohorts, NPS should be used as a complementary tool, rather than replacing, existing ACS risk scores.

Population characteristics such as nutritional status, baseline inflammatory burden, prevalence of cardiometabolic risk factors, and overall dietary patterns vary substantially across countries and may influence NPS components, including serum albumin, cholesterol levels, and leukocyte‐derived ratios [39, 40]. Additionally, regional differences in healthcare delivery, including pre‐hospital care, STEMI networks, door‐to‐balloon times, secondary prevention practices, and access to guideline‐directed therapies, may affect both NPS values and clinical outcomes [41, 42]. As a result, the prognostic performance of NPS observed in this meta‐analysis may not be fully generalizable to international populations since all existing evidence is derived from a single‐country population. Future multicenter, multinational studies are needed to validate these findings across diverse healthcare systems and ethnic groups.

Taken together, these findings suggest the clinical relevance of the NPS as a composite indicator of systemic inflammation and nutritional status, both of which are significant in the pathogenesis and adverse outcomes of ACS. The clinical implementation of NPS is highly feasible, as it is derived from laboratory values that are already part of standard ACS evaluation, enabling rapid, point‐of‐care risk stratification before PCI. Because NPS relies solely on routinely available biomarkers without requiring specialized assays or imaging, it may also serve as a cost‐effective tool for early prognostic assessment, particularly in resource‐limited or high‐volume centers. While formal cost‐effectiveness analyses are lacking, the use of an inexpensive, readily available score that may improve risk stratification is conceptually attractive and supports further evaluation of NPS‐guided strategies in prospective studies.

### 4.1 Limitations

This meta‐analysis has limitations. First, all included studies were observational in nature, which inherently introduces the risk of selection bias, residual confounding, and limited causal inference. Second, because all seven studies were conducted in Turkey, our study population represents a relatively uniform regional cohort, which may limit generalizability. Differences in nutritional profiles, inflammatory markers, comorbidity burdens, and standards of cardiovascular care across countries may influence the prognostic behavior of the NPS. Third, we could not do subgroup analysis due to absence of individual patient data. Fourth, there was high heterogeneity in some outcomes despite performing a leave‐one‐out analysis to address this limitation. Lastly, an important limitation of this meta‐analysis is the substantial variability in follow‐up durations across included studies, ranging from in‐hospital outcomes to as long as 8 years. Such heterogeneity influences the interpretability of mortality outcomes because short‐term in‐hospital mortality captures acute procedural and hemodynamic complications, whereas long‐term mortality reflects chronic disease progression, secondary prevention adherence, and comorbidity burden.

## 5 Conclusion

High NPS was associated with increased all‐cause mortality, higher incidence of no‐reflow, and lower LVEF in ACS patients undergoing PCI. These findings support the prognostic utility of NPS as a simple, accessible risk stratification tool in ACS.

## Author Contributions

Conceptualization: Yasin Özen, Mustafa Bilal Ozbay, Zahin Shahriar. Methodology & Data Extraction: Yasin Özen, Hüseyin Tezcan, Tugay Dedebali. Statistical Analysis: Mustafa Bilal Ozbay, Abdullah Tunçez. Supervision & Critical Review: Kadri Murat Gürses, Bülent Özbay. Manuscript Drafting: Mustafa Bilal Ozbay, Yasin Özen, Zahin Shahriar. Final Approval: All authors read and approved the final manuscript. Guarantor of the review: Mustafa Bilal Ozbay.

## Funding

The authors received no specific funding for this work.

## Ethics Statement

This study is a systematic review and meta‐analysis of previously published studies. No human subjects were directly involved, and ethical approval was not required.

## Conflicts of Interest

The authors declare no conflicts of interest.

## Supporting information

supplementary material_final version.docx.

## Data Availability

All data analyzed in this study are from publicly available published articles. The full dataset used and analyzed during the current study is available from the corresponding author upon reasonable request.

## References

[1] Y. Yang , Z. Liu , F. Gao , et al., “CCC‐ACS Investigators. In‐Hospital Outcomes in Patients With Acute Myocardial Infarction and No Standard Modifiable Cardiovascular Risk Factors Across Varying Body Mass Index: Findings From the CCC‐ACS Project,” Journal of the American Heart Association 14, no. 7 (2025): e037651. 40135554 10.1161/JAHA.124.037651 PMC12132895

[2] N. P. Goodwin , R. M. Clare , J. L. Harrington , et al., “Morbidity and Mortality Associated With Heart Failure in Acute Coronary Syndrome: A Pooled Analysis of 4 Clinical Trials,” Journal of Cardiac Failure 29, no. 12 (2023): 1603–1614. 37479054 10.1016/j.cardfail.2023.07.004

[3] C. J. Vrints , “Non‐ST Elevation Acute Coronary Syndromes: Timing and Selection of Early Invasive Management, ECG Monitoring Need, DAPT Duration, Pathogenesis of Recurrence and Beware of Delirium in the Intensive/Acute Cardiac Care Unit!,” European Heart Journal. Acute Cardiovascular Care 6, no. 6 (2017): 475–476. 28933208 10.1177/2048872617734983

[4] A. De Paiva Fagundes and D. A. Morrow , “Which Patients With Acute Coronary Syndrome Need the Cardiac Intensive Care Unit: Tuning the Tools for Risk Stratification,” European Heart Journal. Acute Cardiovascular Care 9, no. 6 (2020): 543–545. 33203233 10.1177/2048872620963490

[5] E. A. Dziedzic , J. S. Gąsior , A. Tuzimek , et al., “Investigation of the Associations of Novel Inflammatory Biomarkers‐Systemic Inflammatory Index (SII) and Systemic Inflammatory Response Index (SIRI)‐With the Severity of Coronary Artery Disease and Acute Coronary Syndrome Occurrence,” International Journal of Molecular Sciences 23, no. 17 (2022): 9553. 36076952 10.3390/ijms23179553 PMC9455822

[6] A. Tuzimek , E. Dziedzic , J. Beck , and W. Kochman , “Correlations Between Acute Coronary Syndrome and Novel Inflammatory Markers (Systemic Immune‐Inflammation Index, Systemic Inflammation Response Index, and Aggregate Index of Systemic Inflammation) in Patients With and Without Diabetes or Prediabetes,” Journal of Inflammation Research 17 (2024): 2623–2632. 38707954 10.2147/JIR.S454117 PMC11067916

[7] O. Birdal , L. Pay , E. Aksakal , et al., “Naples Prognostic Score and Prediction of Left Ventricular Ejection Fraction in STEMI Patients,” Angiology 75, no. 1 (2024): 36–43. 36863021 10.1177/00033197231161903

[8] Z. Xu , M. Pei , X. Yang , et al., “Associations of Naples Prognostic Score With Stroke in Adults and All Cause Mortality Among Stroke Patients,” Scientific Reports 15, no. 1 (2025): 10718. 40155756 10.1038/s41598-025-94975-2 PMC11953244

[9] M. Czinege , V. B. Halațiu , V. Nyulas , et al., “Nutritional Status and Recurrent Major Cardiovascular Events Following Acute Myocardial Infarction‐A Follow‐up Study in a Primary Percutaneous Coronary Intervention Center,” Nutrients 16, no. 7 (2024): 1088. 38613121 10.3390/nu16071088 PMC11013633

[10] F. Şaylık , T. Çınar , M. Selçuk , T. Akbulut , M. Hayıroğlu , and İ. H. Tanboğa , “Evaluation of Naples Score for Long‐Term Mortality in Patients With ST‐Segment Elevation Myocardial Infarction Undergoing Primary Percutaneous Coronary Intervention,” Angiology 75, no. 8 (2024): 725–733. 37058422 10.1177/00033197231170982

[11] A. Erdogan , O. Genc , E. Ozkan , et al., “Impact of Naples Prognostic Score at Admission on In‐Hospital and Follow‐up Outcomes Among Patients With ST‐Segment Elevation Myocardial Infarction,” Angiology 74, no. 10 (2023): 970–980. 36625023 10.1177/00033197231151559

[12] S. Karakoyun , M. Cagdas , A. I. Celik , et al., “Predictive Value of the Naples Prognostic Score for Acute Kidney Injury in ST‐Elevation Myocardial Infarction Patients Undergoing Primary Percutaneous Coronary Intervention,” Angiology 75, no. 6 (2024): 576–584. 36888971 10.1177/00033197231161922

[13] C. Yılmaz , A. Karaduman , M. M. Tiryaki , et al., “Predictive Value of the Naples Prognostic Score for No‐Reflow Phenomenon After Saphenous Vein Graft Stenting,” Biomarkers in Medicine 19, no. 1 (2025): 13–22. 39711087 10.1080/17520363.2024.2443383 PMC11731040

[14] M. Gitmez , E. Ekingen , and S. Zaman , “Predictive Value of the Naples Prognostic Score for One‐Year Mortality in NSTEMI Patients Undergoing Selective PCI,” Diagnostics 15, no. 5 (2025): 640, 10.3390/diagnostics15050640. 40075887 PMC11899088

[15] M. Saygi , A. C. Tanalp , O. Tezen , et al., “The Prognostic Importance of the Naples Prognostic Score for In‐Hospital Mortality in Patients With ST‐Segment Elevation Myocardial Infarction,” Coronary Artery Disease 35, no. 1 (2024): 31–37. 37990558 10.1097/MCA.0000000000001285

[16] D. F. Stroup , “Meta‐Analysis of Observational Studies in Epidemiology: A Proposal for Reporting. Meta‐Analysis Of Observational Studies in Epidemiology (MOOSE) Group,” Journal of the American Medical Association 283, no. 15 (2000): 2008–2012. 10789670 10.1001/jama.283.15.2008

[17] M. J. Page , J. E. McKenzie , P. M. Bossuyt , et al., “The PRISMA 2020 Statement: An Updated Guideline for Reporting Systematic Reviews,” BMJ 372 (2021): n71. 33782057 10.1136/bmj.n71 PMC8005924

[18] J. A. Sterne , M. A. Hernán , B. C. Reeves , et al., “ROBINS‐I: A Tool for Assessing Risk of Bias in Non‐Randomised Studies of Interventions,” BMJ 355 (2016): i4919, 10.1136/bmj.i4919. 27733354 PMC5062054

[19] J. P. T. Higgins , J. Thomas , J. Chandler , et al. Cochrane Handbook for Systematic Reviews of Interventions Version 6.3 (updated 2022).

[20] Y. Feng , W. Xu , S. Tang , et al., “Inflammation, Nutrition, and Biological Aging: The Prognostic Role of Naples Prognostic Score in Nonalcoholic Fatty Liver Disease Outcomes,” Diabetes Research and Clinical Practice 213 (2024 Jul): 111749. 38906332 10.1016/j.diabres.2024.111749

[21] O. K. Uysal , D. Ozdogru , A. Yildirim , I. Ozturk , G. Tras , and Z. Arlier , “The Prognostic Value of a Naples Score in Determining In‐Hospital Mortality in Patients With Acute Ischemic Stroke Undergoing Endovascular Treatment,” Journal of Clinical Medicine 13, no. 21 (2024): 6434. 39518572 10.3390/jcm13216434 PMC11546944

[22] M. Y. Henein , S. Vancheri , G. Longo , and F. Vancheri , “The Role of Inflammation in Cardiovascular Disease,” International Journal of Molecular Sciences 23, no. 21 (2022): 12906. 36361701 10.3390/ijms232112906 PMC9658900

[23] Y. Zhu , X. Xian , Z. Wang , et al., “Research Progress on the Relationship Between Atherosclerosis and Inflammation,” Biomolecules 8, no. 3 (2018): 80. 30142970 10.3390/biom8030080 PMC6163673

[24] A. A. Manolis , T. A. Manolis , H. Melita , D. P. Mikhailidis , and A. S. Manolis , “Low Serum Albumin: A Neglected Predictor in Patients With Cardiovascular Disease,” European Journal of Internal Medicine 102 (2022): 24–39. 35537999 10.1016/j.ejim.2022.05.004

[25] T. Angkananard , T. Anothaisintawee , M. McEvoy , J. Attia , and A. Thakkinstian , “Neutrophil Lymphocyte Ratio and Cardiovascular Disease Risk: A Systematic Review and Meta‐Analysis,” BioMed Research International 2018 (2018): 2703518, 10.1155/2018/2703518. 30534554 PMC6252240

[26] X. Q. Quan , R. C. Wang , Q. Zhang , C. T. Zhang , and L. Sun , “The Predictive Value of Lymphocyte‐to‐Monocyte Ratio in the Prognosis of Acute Coronary Syndrome Patients: A Systematic Review and Meta‐Analysis,” BMC Cardiovascular Disorders 20, no. 1 (2020): 338. 32669086 10.1186/s12872-020-01614-x PMC7362430

[27] M. Sagris , P. Theofilis , A. S. Antonopoulos , et al., “Inflammation in Coronary Microvascular Dysfunction,” International Journal of Molecular Sciences 22, no. 24 (2021): 13471. 34948272 10.3390/ijms222413471 PMC8703507

[28] L. S. F. Konijnenberg , P. Damman , D. J. Duncker , et al., “Pathophysiology and Diagnosis of Coronary Microvascular Dysfunction in ST‐Elevation Myocardial Infarction,” Cardiovascular Research 116, no. 4 (2020): 787–805. 31710673 10.1093/cvr/cvz301 PMC7061278

[29] L. Back and A. Ladwiniec , “Saphenous Vein Graft Failure: Current Challenges and a Review of the Contemporary Percutaneous Options for Management,” Journal of Clinical Medicine 12, no. 22 (2023): 7118. 38002729 10.3390/jcm12227118 PMC10672592

[30] M. Canu , C. Khouri , S. Marliere , et al., “Prognostic Significance of Severe Coronary Microvascular Dysfunction Post‐PCI in Patients With STEMI: A Systematic Review and Meta‐Analysis,” PLoS One 17, no. 5 (2022): e0268330. 35576227 10.1371/journal.pone.0268330 PMC9109915

[31] Y. Al‐Najjar and A. L. Clark , “Predicting Outcome in Patients With Left Ventricular Systolic Chronic Heart Failure Using a Nutritional Risk Index,” American Journal of Cardiology 109, no. 9 (2012): 1315–1320. 22335857 10.1016/j.amjcard.2011.12.026

[32] A. D. DeVore , A. S. Hellkamp , L. Thomas , et al., “Improvement in Left Ventricular Ejection Fraction in Outpatients With Heart Failure With Reduced Ejection Fraction: Data From CHAMP‐HF,” Circulation: Heart Failure 13, no. 7 (2020): e006833. 32580657 10.1161/CIRCHEARTFAILURE.119.006833

[33] K. Breathett , L. A. Allen , J. Udelson , G. Davis , and M. Bristow , “Changes in Left Ventricular Ejection Fraction Predict Survival and Hospitalization in Heart Failure With Reduced Ejection Fraction,” Circulation: Heart Failure 9, no. 10 (2016): e002962. 27656000 10.1161/CIRCHEARTFAILURE.115.002962 PMC5082710

[34] F. D'Ascenzo , O. De Filippo , G. Gallone , et al., “Machine Learning‐Based Prediction of Adverse Events Following an Acute Coronary Syndrome (PRAISE): a Modelling Study of Pooled Datasets,” Lancet 397, no. 10270 (2021): 199–207. 33453782 10.1016/S0140-6736(20)32519-8

[35] C. Templin and D. Di Vece , “Risk Scores in Predicting Adverse Events Following Acute Coronary Syndrome,” Lancet 397, no. 10270 (2021): 172–173. 33453766 10.1016/S0140-6736(21)00038-6

[36] F. D'Ascenzo , G. Biondi‐Zoccai , C. Moretti , et al., “TIMI, GRACE and Alternative Risk Scores in Acute Coronary Syndromes: A Meta‐Analysis of 40 Derivation Studies on 216,552 Patients and of 42 Validation Studies on 31,625 Patients,” Contemporary Clinical Trials 33, no. 3 (2012): 507–514. 22265976 10.1016/j.cct.2012.01.001

[37] E. M. Antman , M. Cohen , P. J. L. M. Bernink , et al., “The TIMI Risk Score for Unstable Angina/non‐ST Elevation MI: A Method for Prognostication and Therapeutic Decision Making,” Journal of the American Medical Association 284, no. 7 (2000): 835–842. 10938172 10.1001/jama.284.7.835

[38] K. A. A. Fox , G. Fitzgerald , E. Puymirat , et al., “Should Patients With Acute Coronary Disease be Stratified for Management According to Their Risk? Derivation, External Validation and Outcomes Using the Updated GRACE Risk Score,” BMJ Open 4, no. 2 (2014): e004425. 10.1136/bmjopen-2013-004425 PMC3931985 24561498

[39] J. Wang , W. A. Masters , Y. Bai , D. Mozaffarian , E. N. Naumova , and G. M. Singh , “The International Diet‐Health Index: A Novel Tool to Evaluate Diet Quality for Cardiometabolic Health Across Countries,” BMJ Global Health 5, no. 7 (2020): e002120. 10.1136/bmjgh-2019-002120 PMC7375435 32694217

[40] R. Toms , A. Bonney , D. J. Mayne , X. Feng , and R. Walsan , “Geographic and Area‐Level Socioeconomic Variation in Cardiometabolic Risk Factor Distribution: A Systematic Review of the Literature,” International Journal of Health Geographics 18, no. 1 (2019): 1. 30621786 10.1186/s12942-018-0165-5 PMC6323718

[41] J. Park , K. H. Choi , J. M. Lee , et al., “KAMIR‐NIH (Korea Acute Myocardial Infarction Registry–National Institutes of Health) Investigators. Prognostic Implications of Door‐to‐Balloon Time and Onset‐to‐Door Time on Mortality in Patients With ST ‐Segment‐Elevation Myocardial Infarction Treated With Primary Percutaneous Coronary Intervention,” Journal of the American Heart Association 8, no. 9 (2019): e012188. 31041869 10.1161/JAHA.119.012188 PMC6512115

[42] I. Colaiori , G. Biondi‐Zoccai , L. Spadafora , et al., “Regional Disparities in the Management and Outcomes of ST‐Elevation Myocardial Infarction: An Italian Analysis Focusing on Time‐Dependent Reperfusion Networks and In‐Hospital Logistics,” Panminerva Medica 67, no. 1 (2025): 1–9. 39705020 10.23736/S0031-0808.24.05277-7

