# Supplementary material for: Naples Prognostic Score and Clinical Outcomes After PCI for Acute Coronary Syndrome: A Systematic Review and Meta‐Analysis
Source: Clin Cardiol. 2025 Dec 29;49(1):e70247. doi: 10.1002/clc.70247 (PMC12746426; doi:10.1002/clc.70247)
Supplement: Supplementary file 1 — supplementary material_final version.docx. [file CLC-49-e70247-s001.docx]

**Supplemental Material**

**Contents**

[Full search strategy for each database 2](#_Toc199856520)

[Table S1. 3](#_Toc199856521)

[Figure S1. 4](#_Toc199856522)

[Figure S2. 5](#_Toc199856523)

[Figure S3. 6](#_Toc199856524)

[Figure S4. 7](#_Toc199856525)

[Table S2. 8](#_Toc199856526)

# Full search strategy for each database

- PubMed

("NAPLES") AND (PCI OR "percutaneous coronary intervention" OR “Acute Coronary Syndrome"[Mesh] OR "acute coronary syndrome" OR ACS OR "unstable angina" OR "non-ST elevation myocardial infarction" OR NSTEMI OR "ST elevation myocardial infarction" OR STEMI)

- Embase

('naples') AND ('pci' OR 'percutaneous coronary intervention' OR 'acute coronary syndrome'/exp OR 'acute coronary syndrome' OR 'acs' OR 'unstable angina' OR 'non-st elevation myocardial infarction' OR 'nstemi' OR 'st elevation myocardial infarction' OR 'stemi')

- Cochrane Library

("NAPLES") AND (PCI OR "percutaneous coronary intervention" OR "acute coronary syndrome" OR ACS OR "unstable angina" OR "non-ST elevation myocardial infarction" OR NSTEMI OR "ST elevation myocardial infarction" OR STEMI)

Table S1. Naples Prognostic Score (NPS) calculation.

|  | Cut-off value | Score = 1 if... | Score = 0 if... |
| --- | --- | --- | --- |
| Serum Albumin | ≥ 4.0 g/dL | < 4.0 g/dL | ≥ 4.0 g/dL |
| Total Cholesterol | ≥ 180 mg/dL | < 180 mg/dL | ≥ 180 mg/dL |
| Neutrophil-to-Lymphocyte Ratio | ≤ 2.96 | > 2.96 | ≤ 2.96 |
| Lymphocyte-to-Monocyte Ratio | ≥ 4.44 | < 4.44 | ≥ 4.44 |
| Total Naples Score | 0 to 4 | Sum of individual scores |  |
|  |  | **Low NPS: 0–2 points** | **High NPS: 3–4 points** |


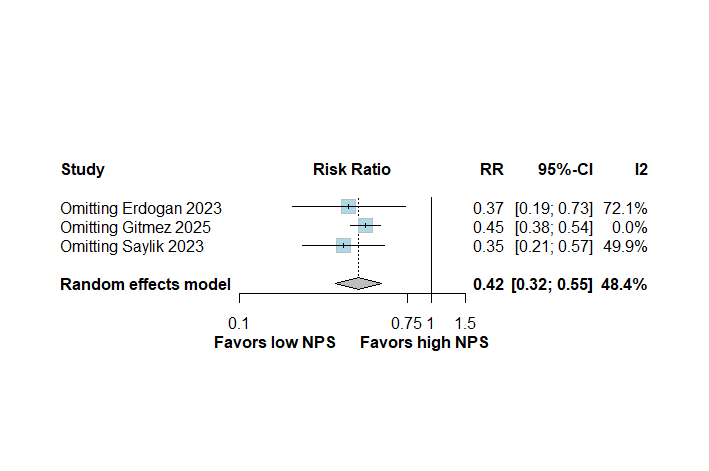


Figure S1. Leave-one-out Analysis for all-cause mortality.


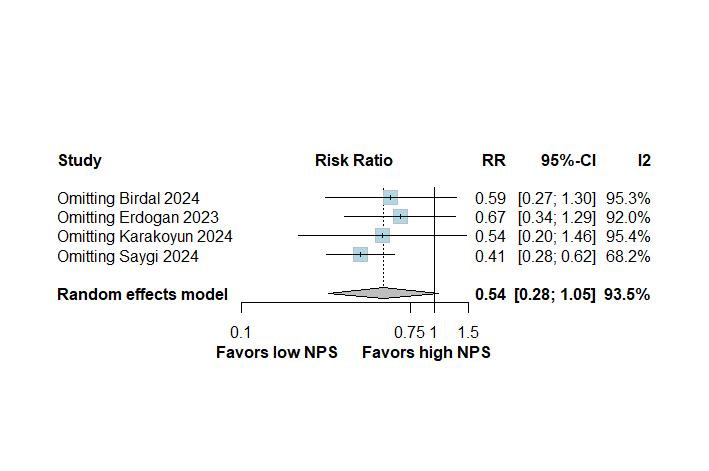


Figure S2. Leave-one-out Analysis for in-hospital mortality.


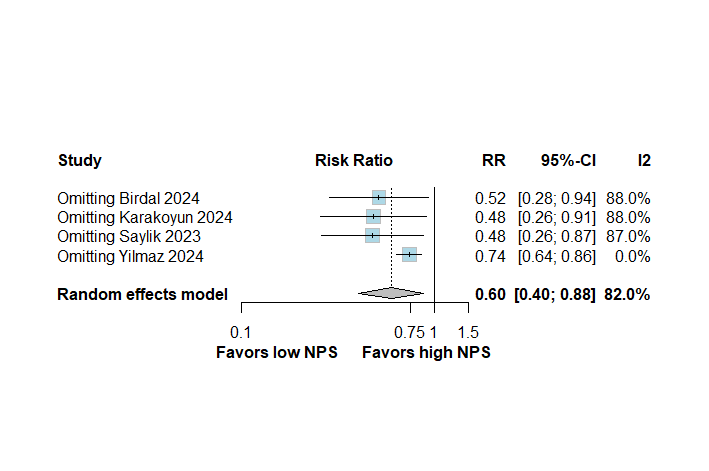


Figure S3. Leave-one-out Analysis for no-reflow.


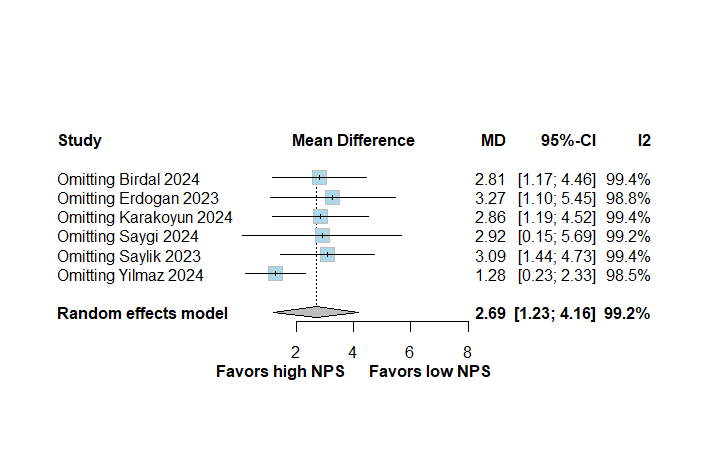


Figure S4. Leave-one-out Analysis for left ventricular ejection fraction (LVEF).

| **Study** | **Bias due to confounding** | **Bias in selection of participants** | **Bias in classification of interventions** | **Bias due to deviations from intended interventions** | **Bias due to missing data** | **Bias in measurement of outcomes** | **Bias in selection of the reported result** | **Overall risk of bias judgement** |
| --- | --- | --- | --- | --- | --- | --- | --- | --- |
| Birdal 2024 | Moderate | Moderate | Low | Low | Moderate | Low | Moderate | Moderate |
| Erdogan 2023 | Moderate | Moderate | Low | Low | Moderate | Low | Low | Moderate |
| Gitmez 2025 | Moderate | Moderate | Low | Low | Low | Low | Low | Moderate |
| Karakoyun 2024 | Moderate | Moderate | Low | Low | Low | Low | Moderate | Moderate |
| Saygi 2024 | Moderate | Low | Low | Low | Low | Low | Moderate | Moderate |
| Saylik 2023 | Moderate | Moderate | Low | Low | Moderate | Low | Moderate | Moderate |
| Yilmaz 2024 | Moderate | Low | Low | Low | Low | Moderate | Moderate | Moderate |

Table S2. Risk of bias summary for non-randomized studies (ROBINS-I) (1).

**Reference**

1. Sterne JA, Hernán MA, Reeves BC, et al. ROBINS-I: a tool for assessing risk of bias in non-randomised studies of interventions. BMJ. 2016;i4919.
